# Role of social media marketing activities in China’s e-commerce industry: A stimulus organism response theory context

**DOI:** 10.3389/fpsyg.2022.941058

**Published:** 2022-08-05

**Authors:** Muhammad Sohaib, Asif Ali Safeer, Abdul Majeed

**Affiliations:** ^1^School of Business Administration, Xi’an Eurasia University, Xi’an, China; ^2^Business School, Huanggang Normal University, Huanggang, China

**Keywords:** social media marketing activities (SMMAs), commitment, trust, satisfaction, online repurchase intentions, E-Commerce

## Abstract

Social media marketing has become one of the most significant growth paths for many businesses in today’s world. However, many companies are still unclear about using social media marketing to get their advantages, particularly in an e-commerce environment. In this background, this study is proposed to examine the effects of social media marketing activities (SMMAs) (interactivity, informativeness, word-of-mouth-WOM, personalization, and trendiness) on relationship quality, such as commitment, trust, and satisfaction in order to predict consumers’ online repurchase intentions in China’s e-commerce environment. This study proposed a theoretical model by using the stimulus-organism-response (S-O-R) theory. Using a structured questionnaire and purposive sampling, this study examined the responses of 403 consumers through partial least square-structural equation modeling. The findings discovered that SMMAs significantly strengthen the relationship quality factors, such as commitment, trust, and satisfaction, which in turn positively increase consumer online repurchase intentions in China’s e-commerce industry. This is novel research that contributes to the S-O-R theory and provides several managerial guidelines that assist managers in improving their business performance in the e-commerce industry. This research also highlighted some limitations.

## Introduction

The internet, social media, mobile applications, and many other virtual communication technologies have permeated billions of people’s daily lives (Yu et al., 2022). Recent statistics revealed that more than 4.60 billion people were active internet users around the world, accounting for around 59.5% of the population in the world. Thus, there were 4.32 billion mobile internet users, and 4.2 billion were active users on social media platforms (Statista, 2022). Therefore, social media has become an integral part of the lives of many individuals around the world. On the other side, western social media platforms are restricted in China, such as Twitter, Facebook, and YouTube are not officially permitted. This prohibition characterizes China’s social media ecology. In recent years, China’s social media users have increased significantly on various social media platforms, including WeChat, Sina Weibo (Chinese Twitter), Little Red Book (XioHongShu, also known as Chinese Instagram), Douyin (Chinese TikTok), QQ (Chinese MSN), and Youku (Chinese YouTube) are prominent among other Chinese social media platforms. As of April 2022, WeChat had 1.26 billion monthly active users, Sina Weibo had 573 million monthly active users, Little Red Book (XioHongShu) had 200 million monthly active users, Douyin had 800 million monthly active users, QQ had 573 million (2020) monthly active users, and Youku had 500 million (2020) monthly active users (Gentlemen Marketing Agency, 2022). Generally, social media gives customers a new way to learn about products and communicate with others worldwide who have had similar experiences with products and services (Yang, 2019). Thus, social media has revolutionized the dynamics of business and marketing, as organizations leverage social media to educate, attract, and retain existing customers (Yang et al., 2022).

Social media is “a group of internet-based applications that build on the ideological and technological foundations of Web 2.0, and that allow the creation and exchange of user-generated content” (Kaplan and Haenlein, 2010, p. 61). Companies can reap significant benefits in the present era by incorporating social media marketing strategies in their business units (Dwivedi et al., 2020, 2021). Companies use social media to interact with consumers, enhance brand awareness, influence consumer behavior, build relationships, and consumer feedback assists them in improving their existing products and services, as well as sales volumes (Lal et al., 2020). On the other hand, social media is increasingly empowering customers and enabling them to take control of the marketing communication process; they are becoming message originators, collaborators, and observers (Hamilton et al., 2016). Thus, it has become important for marketers to strategically exploit social media to gain a competitive advantage and higher performance (Lamberton and Stephen, 2016). In the context of strategic marketing, social media interaction comprises a procedure that enables not only companies to exchange resources but also customers to do so. For instance, Hollebeek et al. (2019) argue that customers can allocate operand (equipment) and operant (knowledge) resources to companies during interactions. With social media’s growing importance in acquiring consumers and market intelligence, marketers may strategically design distinct social media resources based on their existing organizational capabilities and resources (Li et al., 2021).

E-commerce is a growing area that covers the direct and indirect purchasing, selling, and trading of products and services through computer networks worldwide (Gunasekaran et al., 2002). According to Garrett and Skevington (1999), E-commerce encompasses all elements of business, such as ordering, commercial market development, supply chain, and money transfer, by employing new communications technologies. A new era in China’s economy started with the growth of the e-commerce market, which accounted for more than 38% of the country’s GDP (gross domestic product) by 2020. In 2021, China accounted for more than 50% of worldwide e-commerce retail sales, surpassing Europe and the United States combined (Yihan, 2022). According to eMarketer (2020), retail e-commerce sales in China are expected to reach $3.085 trillion by 2022, increasing to $3.331 trillion by 2023 and $3.565 trillion by 2024. China has the world’s most technologically savvy population. In 2019, China had 883 million internet users, more than three times the internet user population in the United States. Similarly, a Statista (2021) survey forecasts that China will have more than 1,069 million social media users by the end of 2022 and more than 1,279 million users by the end of 2026. Thus, it revealed that China has great potential for e-commerce platforms for selling products and services. According to International Trade Administration (2021), China’s e-commerce business is dominated by Alibaba’s Taobao and Tmall (with 50.8% market share), JD.com (with 15.9% market share), and Pinduoduo (with 13.2% market share) become the third-largest platform in China. In recent years, social media platforms like WeChat, TikTok, and Weibo have been enormously popular in China, garnering enormous numbers of domestic and international users. These social media platforms have great potential to attract a larger consumer base, and many companies are striving to leverage these platforms for their marketing activities. For example, Douyin (Chinese TikTok) generated $27.2 billion in revenue from advertisements in 2020 (Zhang et al., 2022). Social media platforms frequently employ novel strategies to facilitate e-commerce. For instance, WeChat enables marketers to target its 1.2 billion users with innovative marketing strategies or “Mini Programs” that allow retailers to showcase online stores and push notifications to promote special offers or launch new product lines. By 2020, WeChat reported doubled revenues ($250 billion) from transactions *via* its “Mini Programs” (International Trade Administration, 2021).

The E-shopping trend has been growing in recent years (Cachero-Martínez and Vázquez-Casielles, 2021), and social media continues to be relevant and trendy due to its interaction and provides enormous possibilities for relationship development. Thus, it is critical to examine social media marketing activities (SMMAs) in e-commerce (Ismail, 2017; Yadav and Rahman, 2018). The growing penetration of social media into society is an effective means of disseminating information and socializing, bringing a new era of e-commerce called social commerce (Zhang et al., 2014). SMMAs are a critical aspect of social commerce practices (Liang and Turban, 2011), which encompass a range of activities such as user reviews, ratings, recommendations, online forums, and e-commerce (Hajli, 2015). Prior research has primarily focused on the SMMAs in the branding context in terms of brand loyalty, brand image, brand awareness, and repurchase intentions in various consumer environments (Ismail, 2017; Cheung et al., 2020, 2021; Ebrahim, 2020; Yang et al., 2022) while few studies have focused on the SMMAs in the e-commerce context in terms of purchase intention, brand equity, and customer loyalty from the Indian perspective (Yadav and Rahman, 2017, 2018). On the other side, many researchers have emphasized the great potential of SMMAs and have urged additional research in various consumer environments (Appel et al., 2020; Dwivedi et al., 2021; Li et al., 2021; Zahay, 2021). In light of this background, this study aims to investigate the impact of SMMAs on consumer online repurchase intentions *via* relationship quality (commitment, trust, and satisfaction) in China’s e-commerce industry. Several scholars acknowledged the significance of relationship quality factors and advocated for additional research, particularly in e-commerce (Yadav and Rahman, 2018). Thus, it is important to answer the following questions in order to achieve the objectives:

What is the impact of SMMAs on relationship quality factors (commitment, trust, and satisfaction) and online repurchase intentions?

How do relationship quality factors (commitment, trust, and satisfaction) affect online repurchase intentions?

This novel research contributes to the theory of stimulus-organism-response (S-O-R) in the context China’s e-commerce business. Further, this study provides several managerial guidelines for developing and executing several business strategies in the online environment of China. In order to organize this study, first, we introduce the topic by highlighting its importance, the research gap, the research objectives, and the research questions. Second, we develop a theoretical research model by following the S-O-R framework, formulate related hypotheses, and design the methodology. Finally, we analyze and discuss the findings, indicating theoretical and managerial implications, concluding remarks, limitations, and future research scope.

## Theoretical framework and hypotheses development

**FIGURE 1:**
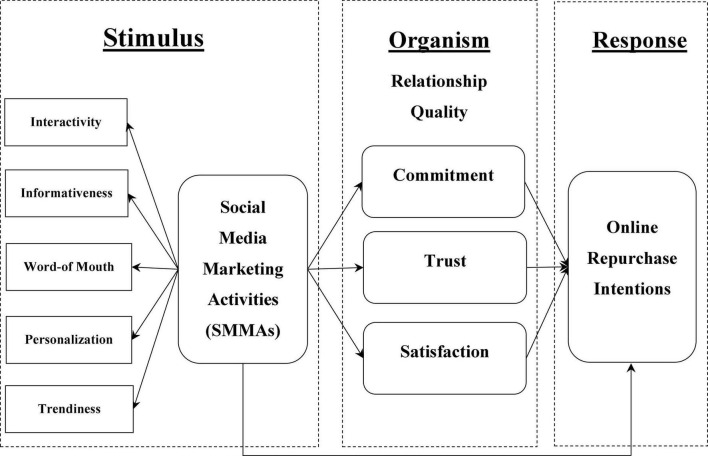
Proposed research model.

The core assumption of this study is that SMMAs (stimulus-S) have a positive effect on consumers’ relationship quality (commitment, trust, and satisfaction) (organism-O) to predict consumers online repurchase intentions (response-R). Figure 1 illustrates the theoretical model.

Drawing from the literature, we propose that the S-O-R theory is the best fit for the proposed model. Mehrabian and Russell (1974) presented the S-O-R theory and demonstrated that environmental cues (stimuli) can stimulate a person’s self-assessment state (organism), which leads to negative or positive behavior (response). The S-O-R theory asserts that different environmental attributes serve as stimuli, affecting the mental (psychological) condition of persons/organisms and driving them to react behaviorally (Jacoby, 2002; Sohaib et al., 2022). Stimulus refers to an external environmental element that can affect an organism’s cognitive and mental states (Lin and Lo, 2016). Prior research argued that SMMAs may serve as an external environmental stimuli (Koay et al., 2020). Similarly, following a series of involved cognitive processes, the organism would respond to environmental stimuli with either an external or internal behavioral response (Liu and Zheng, 2019; Attiq et al., 2022). The external response takes the form of the individual’s unique behavior, while the internal response takes the form of the individual’s attitude (Lorenzo-Romero et al., 2016). Thus, relationship quality, such as commitment, trust, and satisfaction, could be an organism that drives consumer behavior (Izogo et al., 2017; Pyo, 2020). In addition, the organism’s behavior is not a passive process that involves stimulus and reaction but rather an intentional response to external stimulus. It is the process by which environmental stimuli influence the person’s emotional or cognitive experience and subsequently result in behavioral responses to those stimuli *via* a sequence of inner psychological actions (Hu et al., 2016; Zhu et al., 2020b). Previous research argued that consumers responses could be expressed in the form of repurchase intention (Zhu et al., 2020a). In accordance with the S-O-R theory, this study considered online repurchase intentions as consumer responses in the context of e-commerce business.

The S-O-R theory is comparable to the information processing model. It concentrates on how consumers’ cognitive systems process input from the decisions environment and how that processing results in a final reaction (Wang and Chang, 2013). Prior research has used the S-O-R theory in the e-retail context and discovered that e-retail environmental cues affect consumers’ internal states, which in turn influences their behavior toward the e-retail platform (Eroglu et al., 2003). Similarly, several studies examined consumer behavior in e-commerce environments using the S-O-R theory (Zhang et al., 2014; Ismail, 2017; Yadav and Rahman, 2018). Thus, prior studies have acknowledged the importance and relevance of the S-O-R theory in explaining consumers’ inner conditions and behavioral responses toward online environment stimuli. As a result, the S-O-R theory provides a systematic framework for assessing the effects of SMMAs as external environmental stimuli on relationship quality including commitment, trust, and satisfaction (as organism) to predict consumers’ online repurchase intentions (as consumer response) in the context of China’s e-commerce industry.

### Social media marketing activities and relationship quality (commitment, trust, and satisfaction)

The term “social media marketing” (SMM) has been described differently in the literature, like as a way of connecting and interacting with current and potential customers to develop relationships with them (Chan and Guillet, 2011; Choi et al., 2016). Other scholars characterized it as the practice of increasing the value of stakeholders by incorporating social media platforms into marketing operations (Pham and Gammoh, 2015; Felix et al., 2017). SMMAs (such as interactivity, informativeness, personalization, word of mouth (WOM), and trendiness) are defined as the process by which companies generate, communicate, and disseminate online marketing offers (products and services) *via* social media platforms to establish and maintain relationships that create value for stakeholders (Yadav and Rahman, 2017). Here, interactivity is defined as the degree to which consumers perceive that e-commerce’s social media platforms enable them to communicate content and opinions with the business and other consumers (Yadav and Rahman, 2018). It is essentially a means of interactive communication between companies and consumers (Gallaugher and Ransbotham, 2010). Consumers contribute to businesses’ social media networks to connect with other consumers and discuss different products and services on e-commerce platforms (Muntinga et al., 2011).

Informativeness can be described as the extent to which consumers perceive social media platforms to provide accurate, helpful, and accurate information about e-commerce businesses. Online shoppers frequently make purchasing decisions based on sufficient and reliable information available on e-commerce websites or social media platforms in the form of product details, ratings, and reviews (Yadav and Rahman, 2018). Thus, consumers are motivated to gather comprehensive and relevant information about a particular product *via* e-commerce social media (Kim et al., 2010). Personalization is described as a consumer’s perception of the extent to which an e-commerce website’s social media platforms offer customized services to meet the needs and preferences of consumers. By customizing e-commerce’s social media platforms, businesses may provide a more personalized experience, increase brand loyalty, and build relationships with consumers toward e-commerce platforms (Martin and Todorov, 2010). WOM is defined as consumers’ perceptions of the extent to which they recommend and share their experiences *via* social media regarding e-commerce businesses. Further, it has been characterized as an informal discussion addressed to other consumers regarding product use and ownership (Berger, 2014). WOM has a significant impact on the trust and purchasing behavior of consumers. Similarly, online ratings/reviews are an important source of WOM for e-commerce websites and have influenced and helped consumers to make better purchase decisions (Duan et al., 2008). The term “trendiness” refers to a consumer’s perception of the degree to which an e-commerce site’s social media platforms feature trendy information. Social media platforms provide up-to-date information and breaking news (Naaman et al., 2011). On the other side, “trendy information on social media covers four sub-motivations: surveillance, knowledge, pre-purchase information, and inspiration” (Muntinga et al., 2011). Thus, trends assist companies in attracting consumers to e-commerce sites.

Prior studies primarily focused on SMMAs examined various concepts in diverse consumer environments. For example, the impact of SMMAs was examined on brand equity in India and Egypt (Yadav and Rahman, 2017; Ebrahim, 2020), value and brand consciousness in Malaysia (Ismail, 2017), customer equity drivers in India (Yadav and Rahman, 2018), consumer brand engagement in Hong Kong (Cheung et al., 2020), consumer brand-related activities (consuming, contributing, and creating) in China (Cheung et al., 2021) and brand image and brand awareness in China (Yang et al., 2022), and consumers’ engagement intention and engagement behavior in Pakistan (Shang et al., 2022). Previous research has acknowledged the importance of SMMAs and relationship quality (commitment, trust, and satisfaction) in e-commerce (Yadav and Rahman, 2018). Several authors acknowledged that relationship quality is a comprehensive measure of relationships that are generally considered to be composed of three factors, including commitment, trust, and satisfaction (Hennig-Thurau et al., 2002; Hajli, 2014a). Using social media platforms such as Facebook, Sharma et al. (2020) demonstrated that SMMAs help the companies in strengthening their customer brand relationships for apparel brands. Similarly, SMMAs help companies (leather industry) in increasing consumer brand commitment (Nobar et al., 2020). Consumer trust is critical, especially in the online shopping environment, where buyers cannot physically interact with the products (Haque and Mazumder, 2020). Further, the firm’s reputation in social commerce activities also affects consumer trust (Yahia et al., 2018). Likewise, social media usage and companies online convenient operations enhance consumer satisfaction (Zhan et al., 2016; Duarte et al., 2018). On the other hand, universities benefit from social media engagement by strengthening their relationships with students in terms of commitment, trust, and satisfaction (Clark et al., 2017). According to S-O-R theory, SMMAs as environmental stimuli (stimulus) positively impact individuals’ cognitive and emotions (organism) by strengthening their quality relationships, such as commitment, trust and satisfaction. Thus, we can predict that SMMAs (interactivity, Informativeness, WOM, customization, and trendiness) will assist companies in building positive quality relationships with Chinese consumers by enhancing their commitment, trust, and satisfaction from an e-commerce perspective. Therefore, we can assume the following hypotheses:

H1. SMMAs positively affects commitment.

H2. SMMAs positively affects trust.

H3. SMMAs positively affects satisfaction.

### Social media marketing activities and online repurchase intentions

Chiu et al. (2009) defined “repurchase intention is the subjective probability that an individual will continue to purchase products from the online vendor or store in the future.” Prior research has primarily examined the effect of SMMAs on brand loyalty in India and Egypt (Ismail, 2017; Ebrahim, 2020) and purchase intention in India and Egypt (Yadav and Rahman, 2017; Ibrahim et al., 2020). Similarly, previous research reported positive relationships between SMMAs and online purchase intention toward luxury brands in Korea (Kim and Ko, 2010), the Indian fashion luxury industry (Gautam and Sharma, 2017) and social media user intentions in Pakistan (Jamil et al., 2022). The third component of the S-O-R theory is consumer response (i.e., purchase/repurchase intention) (Zhu et al., 2020b; Yang et al., 2022). The consumer responses serve as the basis for the current investigation on online repurchase intention, also known as consumer online repurchase intention. Previous research has rarely examined the impact of SMMAs on consumer online repurchase intentions in China’s e-commerce business. Thus, we anticipate that SMMAs will positively affect consumer online repurchase intentions. Therefore, we assume:

H4. SMMAs positively affect online repurchase intentions.

### Relationship quality (commitment, trust, and satisfaction) and online repurchase intentions

The concept of relationship quality is very important in relationship marketing. According to De Wulf et al. (2001), relationship quality is primarily determined by three factors: commitment, trust, and satisfaction. Other research has also emphasized the importance of quality relationships (commitment, trust, and satisfaction) in the virtual environment of social commerce (Hajli, 2014a). Thus, this research considered the quality relationships including commitment, trust, and satisfaction as important factors in current investigation. The term “relationship commitment” refers to a person’s willingness to continue purchasing from a particular retailer (De Wulf et al., 2001). Commitment is the most critical aspect of maintaining a long-term relationship (Garbarino and Johnson, 1999). The relationship quality indicators demonstrate the critical nature of the relationship with the service provider by revealing how much effort is expended to ensure the relationship is maintained (Gustafsson et al., 2005). Trust is critical in today’s uncertain online environment, mainly when users perform social and commercial transactions with online merchants (Paul, 2003). Similarly, trust is vital in today’s world of social commerce (Hajli, 2014b). Trust in a business demonstrates that the e-vendor is trustworthy and benevolent (Gefen et al., 2003). Likewise, trustworthy and benevolent are perceived as distinct forms of trust. These attributes increase consumer trust in vendor product information and transactions in an online context, assisting in strengthening buyer-vendor relationships in the online environment (Ba and Pavlou, 2002). The term relationship satisfaction relates to an individual’s affective state as a result of their comprehensive assessment of their relationship with other persons (De Wulf et al., 2001). Investments in customer relationships have a significant impact on consumers’ purchase intentions and retentions (Palmatier et al., 2006). Relationship marketing revolves around the concept of relationship strength or relationship quality (Rauyruen and Miller, 2007). This relationship depends upon the vendor’s service quality and the manner in which it establishes a relationship with consumers (Hajli, 2014a). Previous research emphasizes the value of quality relationships (commitment, trust, and satisfaction) and indicated that it may effect consumer buying behavior. For example, Verhoef et al. (2002) discussed that trust, satisfaction and affective commitment are associated with customer referrals and services purchased. They discovered that trust, satisfaction and affective commitment significantly influenced customer referrals. Similarly, affective commitment positively influenced the number of services purchased. Likewise, Sharma et al. (2020) demonstrated that quality relationships, including trust, satisfaction, and commitment positively impact consumer purchase intentions toward fashion apparel products in the Indian context. Similarly, Sohaib (2022) revealed that relationship quality factors such as, commitment, trust, and satisfaction significantly increase customer repurchase intentions in the banking sector of Pakistan. In general, it is believed that a relationship’s (or its constituents’) high quality results in a proportionally high level of purchase intention (Kuo et al., 2009; Hajli, 2014a). Thus, based on the literature, we predict that the relationship quality (commitment, trust, and satisfaction) will have a significant effect on consumers’ online repurchase intentions in China’s e-commerce context. As a result, we assume:

H5. Commitment positively influences consumer online repurchase intentions.

H6. Trust positively influences consumer online repurchase intentions.

H7. Satisfaction positively influences consumer online repurchase intentions.

## Materials and methods

This study used the social media marketing attributes in China’s e-commerce due to several reasons. First, China has the world’s largest e-commerce market, with the highest retail sales (eMarketer, 2020). Second, China has the world’s biggest online purchasing population, with over 780 million persons (Alexander Ayertey, 2021). Third, there is a dearth of research on this topic in the literature, particularly from the Chinese perspective, and several authors have called for more research (Yadav and Rahman, 2018). A structured questionnaire was designed to obtain data from the target audience. We followed the Yadav and Rahman (2018) following criterion in order to ensure that all consumers were active users of SMM in China’s e-commerce context:

Each user should regularly use social media (WeChat, Weibo, Douyin, etc.);

Each user must have an account with one or more e-commerce apps/sites [e.g., Taobao, Tmall, Jingdong (JD), Pinduoduo, etc.] and make regular purchases from these apps/sites or *via* their product links available on WeChat, Weibo, Douyin, and other social media platforms.

After making purchases from e-commerce apps/sites, provide ratings, comments, and recommendations regarding products and/or referring to them before confirming any new purchase (either on the e-commerce apps/websites or on social media platforms like WeChat, Weibo, Douyin, etc.);

Promote products of various e-commerce apps/sites [e.g., Taobao, Tmall, Jingdong (JD), Pinduoduo, etc.] through social media platforms (like WeChat, Weibo, Douyin, etc.); and

How frequently do you purchase products through e-commerce apps/sites?

The abovementioned criteria were strictly adhered in order to ensure that only qualified and relevant participants were included. The questionnaire was prepared in Chinese language and posted on a leading survey website of China.^1^ The survey respondents were asked to randomly select an e-commerce app/site and respond to survey questions based on their opinions. This study used purposive sampling because researchers use purposive sampling to collect data from their intended audience based on their subjective judgment (Sekaran and Bougie, 2016). Many consumers, including students and people of other professions were approached *via* WeChat for data collection. However, students who participated in this survey were in large portion because they are active social media users and technologically savvy (Ismail, 2017). China has the most social media users worldwide (more than 1.2 billion monthly active social media users) (Gentlemen Marketing Agency, 2022). This study primarily collected data from Chinese students, who frequently use e-commerce apps/sites for online product purchases. In order to generalize the findings, we did not specify the products or brands but included users/consumers who buy all types of products through e-commerce apps/sites. The significance of this generalized study’s findings may assist managers in taking a holistic approach while establishing and maintaining e-commerce apps/sites, regardless of the product or brand category (Yadav and Rahman, 2018). We collected 447 responses and, following a rigorous screening process, selected 403 responses for final data analysis (see Table 1 for targeted audience information).

**TABLE 1:** Targeted audience information.

	Number	%
No. of participants	403	100.00%
**Gender**		
Male	186	46.15%
Female	217	53.85%
**Age range**		
18–22	90	22.33%
23–27	174	43.18%
28–32	97	24.07%
33–37	42	10.42%
**Education level**		
Bachelor	108	26.80%
Master	219	54.34%
Doctoral	55	13.65%
Other professional degree	21	5.21%
**Profession**		
Student	289	71.71%
Private and government organizations employee	70	17.37%
Self-own business	31	7.69%
Unemployed	13	3.23%
**Monthly family income (RMB)**		
Up to ¥7,000	162	40.20%
¥7,001– ¥12,000	111	27.54%
¥12,001– ¥17,000	59	14.64%
¥17,001–¥22,000	56	13.90%
More than ¥22,000	15	3.72%
**Social media users’ online shopping**		
Up to 2 times weekly	133	33.00%
3–5 times fortnightly	175	43.42%
More than 5 times monthly	95	23.57%

This research incorporated established scales from prior research studies. We picked 15 questions (3 each for interactivity, informativeness, WOM, personalization, and trendiness) to evaluate SMMAs. These questions were adapted from prior studies on SMM and e-commerce (Yadav and Rahman, 2017, 2018). This study used nine questions (three each of commitment, trust, and satisfaction) to evaluate relationship quality from Hajli (2014a). Finally, this study modified three questions of online repurchase intentions from Wen et al. (2011). All questions were modified in the current research context and respondents rated the questions according to a seven-point Likert scale.

This study measured SMMAs as a higher-order reflective construct. SMMAs were measured using a two-stage disjoint (Becker et al., 2012). This method guides scholars to save the average scores of constructs by using their lower-order components. As a result, we applied this method and saved the average scores in order to quantify SMMAs as a higher-order reflective construct (Safeer et al., 2021b,d). We calculated the average scores of lower-order components (i.e., interactivity, informativeness, WOM, personalization, and trendiness). We measured the SMMAs in the proposed model using the two-stage method with mode A (Sarstedt et al., 2019).

## Results

This study employed the PLS-SEM (partial least square structural-equation modeling technique) technique and used the SmartPLS 3 to evaluate the proposed model. Partial least square is a variance-based structural equation modeling and causal inference technique that has attracted the attention of many researchers in marketing and consumer behavior research in offline and online environments (Cheung et al., 2021; Safeer et al., 2021c,^2022^). Without enforcing data distribution constraints, the PLS-SEM approach assists scholars in managing complex models with several concepts, items, and structural paths (Hair et al., 2019). PLS-SEM is an optimization technique for predicting endogenous constructs (Hair et al., 2017). Numerous scholars recommended evaluating the model in two stages, including measurement and structural model evaluation (Sarstedt et al., 2017; Hair et al., 2018).

Although PLS-SEM can perform data analysis on non-normal data, however, extremely non-normal data may mislead the results (Hair et al., 2017). As a result, we meticulously analyzed the data before conducting the model measurement evaluation and deleted many biased/suspicious responses (i.e., straight-lining responses). We did not discover any missing values due to the online questionnaire’s imposed restrictions. Finally, we evaluated the data for normality using the kurtosis and skewness methods and observed that several values were not normal. Thus, SMART PLS software was the best choice for data analysis.

Before conducting data analysis, it is also necessary to consider common method bias (CMB) in the data. We applied the full collinearity test (Kock, 2015) and analyzed all proposed constructs’ VIF (variance inflation factor) values. We discovered that all VIF values were less than 3.30 based on a recommended threshold (Kock and Lynn, 2012; Kock, 2015). Further, Hair et al. (2017) recommended that VIF values less than 5 indicated no multicollinearity in the data. This method is commonly applicable in consumer behavior research (Qalati et al., 2021; Safeer et al., 2021d). Thus, we believe that our data was free from any potential threat or bias before performing the analysis.

### Measurement model evaluation

By following several authors’ recommendations for model measurement evaluation (Hair et al., 2017; Hair et al., 2019), this study analyzed the constructs AVE (average variance extracted), CR (composite reliability), Cronbach’s alpha, and outer loading values in order to evaluate the proposed model. We discovered that all AVE, CR, Cronbach’s alpha, and outer loading values were within the threshold range (see Table 2) (Hair et al., 2018).

**TABLE 2:** Constructs loading, Cronbach’s Alpha, CR, and AVE values.

	Items	Loading	Cronbach’s alpha	CR	AVE
SMMAs	INF	0.89	0.92	0.94	0.76
	INT	0.83			
	PER	0.89			
	TRD	0.88			
	WOM	0.87			
CMT	CMT1	0.93	0.91	0.94	0.85
	CMT2	0.91			
	CMT3	0.92			
TRT	TRT1	0.94	0.93	0.95	0.87
	TRT2	0.93			
	TRT3	0.93			
SAT	SAT1	0.92	0.91	0.95	0.85
	SAT2	0.93			
	SAT3	0.92			
ORPI	ORPI1	0.93	0.91	0.94	0.85
	ORPI2	0.92			
	ORPI3	0.92			

SMMAs, Social media marketing activities; CMT, Commitment; TRT, Trust; SAT, Satisfaction; ORPI, Online Repurchase intentions.

This research evaluated the discriminant validity of the proposed model using the Fornell-Larcker Criterion (Fornell and Larcker, 1981) and the Heterotrait-Monotrait Ratio (Henseler et al., 2015). The findings revealed that both methods satisfied the recommended threshold values (Fornell and Larcker, 1981; Henseler et al., 2015). Thus, this study successfully established the proposed model’s discriminant validity (see Tables 3a,b).

**TABLE 3a:** Fornell-Larcker criterion.

	CMT	ORPI	SAT	SMMA	TRT
**CMT**	0.92				
**ORPI**	0.63	0.92			
**SAT**	0.81	0.62	0.92		
**SMMA**	0.73	0.52	0.75	0.87	
**TRT**	0.80	0.64	0.78	0.74	0.93

**TABLE 3b:** Heterotrait-Monotrait ratio.

	CMT	ORPI	SAT	SMMA	TRT
**CMT**					
**ORPI**	0.70				
**SAT**	0.89	0.68			
**SMMA**	0.79	0.56	0.81		
**TRT**	0.88	0.69	0.84	0.79	

### Structural model assessment

**FIGURE 2:**
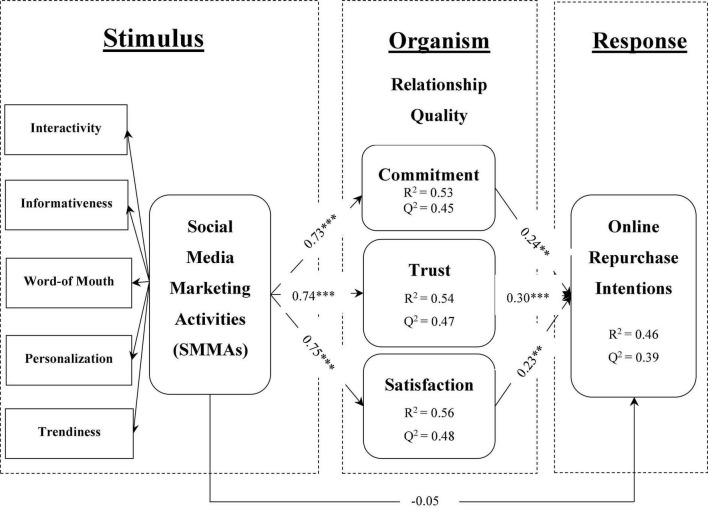
Structural model significance, *R*^2^ and *Q*^2^-values. (*t* > 2.58 at **p < 0.01); (*t* > 3.29 at ***p < 0.001); (two-tailed).

**FIGURE 3:**
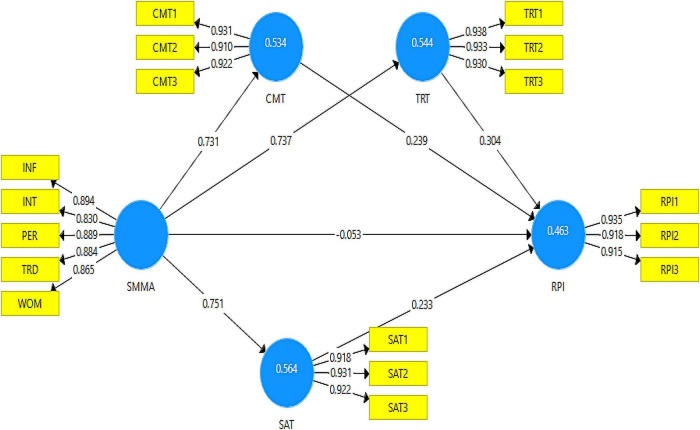
Proposed model relationships and loading values.

Numerous researchers recommended several tests to successfully examine the structural model, including model fit, coefficient of determination (*R*^2^), *Q*^2^ predictive relevance, and model path coefficients (Sarstedt et al., 2017; Hair et al., 2018; Hair et al., 2019). PLS-SEM does not have a perfect fit measure; however, when using the PLS-SEM analysis technique, SRMR can be used to evaluate the proposed model (Hair et al., 2017). This study discovered an excellent model fit, with an SRMR value of 0.04, compared to the recommended value of 0.08 (Hu and Bentler, 1999). *R*^2^ quantified the contribution of exogenous constructs to endogenous constructs. Thus, all *R*^2^-values should be between 0 and 1, with a higher value indicating a larger contribution to endogenous constructs (Hair et al., 2019). This study discovered that exogenous constructs explained 53.40% variance in CMT (commitment), 54.40% variance in TRT (trust), 56.40% variance in SAT (satisfaction), and 46.30% variance in ORPI (online repurchase intentions), indicating a significant contribution to endogenous constructs (Hair et al., 2017). We used the blindfolding procedures to determine the proposed model’s predictive relevance (*Q*^2^) (Geisser, 1974; Stone, 1974). This study discovered that the *Q*^2^-value of CMT (commitment) was 44.90%, TRT (trust) was 47.10%, SAT (satisfaction) was 47.60%, and ORPI (online repurchase intentions) was 38.80%. Hence, predictive relevance of empirical model and measurement model have been achieved (see Figures 2, 3 respectively) (Hair et al., 2019).

To examine the proposed hypotheses, we used the bootstrapping method with 5,000 subsamples bias-corrected and accelerated, two-tailed at the significance level of 0.05 (Chin, 1998; Hair et al., 2017). The findings revealed that SMMAs significantly influenced CMT, TRT, and SAT. Thus, H1–H3 were supported. However, SMMAs did not influence ORPI, such as SMMAs - > ORPI (β = −0.05; *p* = 0.62). Therefore, H4 was not supported. On the other side, CMT, TRT, and SAT positively influenced ORPI, indicating that H5–H7 were supported. This study examined the mediation effects by evaluating relationships directly and indirectly (Hair et al., 2017). The findings discovered that quality relationships (CMT, TRT, and SAT) fully mediated the relationships between SMMAs and ORPI. These quality relationships are essential for developing consumers’ online purchase intentions in the e-commerce context. Overall the proposed model was significant (indirectly), such as SMMAs - > ORPI (β = 0.57; *p* = 0.00). As a result, the findings revealed that the proposed model significantly contributed to SMMAs in the e-commerce context (see Table 4).

**TABLE 4:** Hypotheses testing results.

Hypothesis	Relationships	β-value	*t*-value	Support	
**Direct relationships**					
H1	SMMAs - > CMT	0.73***	21.56	Yes	
H2	SMMAs - > TRT	0.74***	22.52	Yes	
H3	SMMAs - > SAT	0.75***	26.11	Yes	
H4	SMMAs - > ORPI	−0.05	0.50	No	
H5	CMT - > ORPI	0.24**	2.74	Yes	
H6	TRT - > ORPI	0.30***	3.49	Yes	
H7	SAT - > ORPI	0.23**	2.91	Yes	
**Mediating relationships**		**Indirect**	**Direct**	**Mediation**	**Total effects**
SMMAs - > CMT - > ORPI		0.17**	−0.05	Full	0.12
SMMAs - > TRT - > ORPI		0.22***	−0.05	Full	0.17
SMMAs - > SAT - > ORPI		0.17**	−0.05	Full	0.12
**Model effects**					
SMMAs - > ORPI		0.57***	−0.05		0.52

“(t > 2.58 at **p < 0.01); (t > 3.29 at ***p < 0.001); (two-tailed).”

### Importance-performance map analysis

**FIGURE 4:**
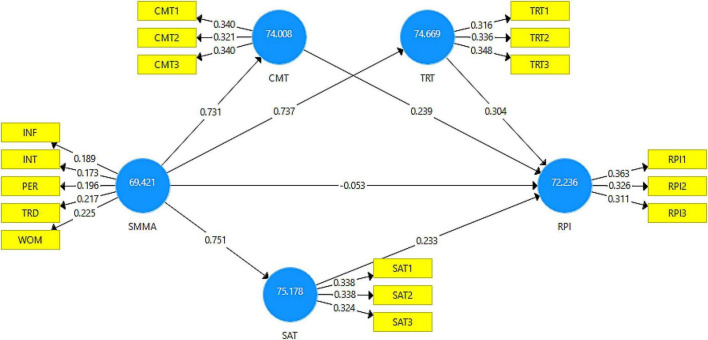
IPMA results presentation.

**FIGURE 5:**
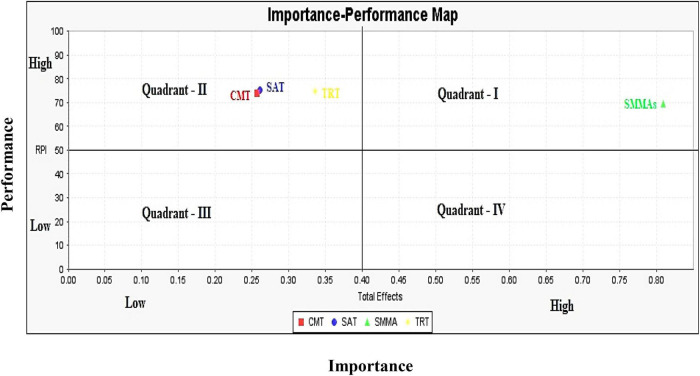
IPMA performance and importance analysis.

By using importance-performance map analysis (IPMA), PLS-SEM findings of path coefficient estimations can be extended by taking into account the average latent variable scores (Hock et al., 2010; Hair et al., 2017). Specifically, the IPMA analyzes the total effects of the structural model on a particular target construct with the average latent variable scores of this target’s antecedents (Ringle and Sarstedt, 2016). The total effects reveal the importance of the antecedent constructs in forming the target construct, whereas the average scores of latent variables demonstrate their performance (Hair et al., 2017). In a practical sense, the IPMA assists corporate management in making smarter decisions (Streukens et al., 2017). The results revealed (see Figure 4) that the scores for the exogenous constructs were greater than 50, such as the SMMAs (69.42), the CMT (74.01), the TRT (74.67), and the SAT (75.18). Likewise, the score for the endogenous construct was greater than 50, such as RPI (72.24). However, the performance and importance may differ due to the variation of these scores. We demonstrate their performance and importance with the help of Figure 5.

Figure 5 displayed four quadrants with high and low importance-performance scores. The findings revealed that SMMAs contributed to high performance and high importance for RPI (i.e., online repurchase intentions) in quadrant—I, whereas CMT, TRT, and SAT contributed to high performance with low importance in quadrant—II. Thus, it demonstrates that Chinese consumers considered SMMAs highly important for their online repurchase intentions, whereas quality relationships, such as CMT, TRT, and SAT were less important to them. The findings assist corporate management in designing online strategies for strengthening quality relationships with Chinese consumers by enhancing their commitment, trust, and satisfaction and should emphasis on moving these important factors to quadrant—I.

## Discussion

This study showed interesting findings. First, SMMAs (interactivity, informativeness, WOM, personalization, and trendiness) significantly strengthen the relationship quality [commitment (H1), trust (H2), and satisfaction (H3)] in China’s e-commerce environment. Several scholars corroborate our findings in different consumer settings, such as Sano (2015) discovered that SMMAs (interaction, trendiness, customization, and risk) significantly increase customer commitment, facilitating service providers to develop long-term relationships with their customers, particularly in the indemnity insurance service. Thus, this study contributed to the literature by examining the effect of SMMAs on commitment (as a relationship quality factor) in China’s e-commerce industry. Similarly, several studies discovered the favorable effects of SMMAs (entertainment, interaction, WOM, trendiness, and customization) on brand trust in various consumer settings, such as in the banking sector of Bangladesh (Hafez, 2021), telecommunication companies of Egypt (Ebrahim, 2020), and hotel industry of Turkey (Tatar and Eren-Erdoǧmuş, 2016). Trust is critical in online transactions, particularly in developing meaningful relationships with customers in social commerce (Hajli, 2014a). Thus, our findings contributed that SMMAs can assist e-commerce businesses in developing high-quality relationships by increasing consumer trust in the online environment. On the other side, Sharma et al. (2020) discovered that SMMAs (interactivity, informativeness, WOM, personalization, and trendiness) significantly strengthened consumer brand relationships in the Indian context by increasing consumer trust, satisfaction, and commitment toward apparel retail brands.

Second, this study did not find any significant impact of SMMAs on consumers’ online repurchase intentions. However, there is scarce evidence to support online repurchase intentions in the current context. As a result, we will encourage scholars to conduct additional research on this construct in order to generalize the findings in an e-commerce environment. Finally, the relationships quality [commitment (H5), trust (H6), and satisfaction (H7)] significantly improved the consumer online repurchase intentions in the e-commerce context. These findings are supported by several studies, such as Elbeltagi and Agag (2016) have demonstrated that customer commitment and satisfaction increase online repurchase intentions in the Egyptian E-retailing context. Similarly, Shin et al. (2013) demonstrated that depending on site quality (site design, shopping convenience, information usefulness, payment system, transaction security, and customer communication), customer trust, satisfaction, and commitment can increase Students’ online repurchase intentions in the Korean context. Thus, this research contributes to the literature by revealing that, depending upon SMMAs, the quality of relationships such as commitment, trust, and satisfaction can increase consumers’ online repurchase intentions in China.

### Theoretical contributions

This study contributes in several ways. First, this study validated the SMMAs scale by using updated components such as interactivity, informativeness, WOM, personalization, and trendiness (Yadav and Rahman, 2017) in the Chinese context. Second, this study examined the impact of SMMAs (interactivity, informativeness, WOM, personalization, and trendiness) on consumer online repurchase intentions *via* relationship quality (commitment, trust, and satisfaction) in China’s e-commerce environment. Prior research highlighted the importance and the gap in relationship quality (commitment, trust, and satisfaction) in the literature (Yadav and Rahman, 2018). Further, such type of research is rarely examined in China’s environment. In recent years, many scholars have focused on the concept of relationship quality (commitment, trust, and satisfaction) (Akrout and Nagy, 2018; Wu et al., 2019) because consumers are highly concerned about their relationship quality issues, such as trust, satisfaction, and commitment in the virtual environment (Nadeem et al., 2020). Finally, this study contributes to stimulus organism response theory (Mehrabian and Russell, 1974) by validating the proposed model. Prior research primarily contributed to the S-O-R theory by investigating customer equity and loyalty in e-commerce (Yadav and Rahman, 2018), brand image, brand awareness, and repurchase intention in high tech products (Yang et al., 2022) within the context of SMMAs. Thus, we examined SMMAs, such as external environmental stimuli (SMMAs) affect consumers’ mental conditions/organisms (relationship quality), thereby triggering behavioral reactions (responses) (Jacoby, 2002; Lorenzo-Romero et al., 2016).

### Managerial contributions

This research presents important managerial recommendations that assist managers in developing diverse marketing strategies for China’s e-commerce industry.

First, this study recognized that several SMMAs, such as interactivity, informativeness, WOM, personalization, and trendiness, can be applied in the Chinese e-commerce industry. A comprehensive understanding of SMMAs may assist managers and marketers in implementing necessary improvements to social media features on e-commerce apps/sites, as well as their activity on other major platforms (WeChat, Weibo, Douyin, etc.). For example, interactivity and informativeness support managers in boosting consumer and company interactions by providing more relevant and customized information to consumers, thereby improving their e-commerce effectiveness. Likewise, personalized and trendy products encourage consumers to share positive WOM.

Second, by examining the impact of SMMAs on relationship quality (commitment, trust, and satisfaction), the managers can strengthen consumers’ trust, satisfaction, and commitment in the e-commerce industry. For example, managers can build consumer trust by enhancing consumer interactivity and delivering reliable information. Similarly, customized and trendy products can drive consumers to promote positive WOM by increasing their commitment and satisfaction. Consequently, SMMAs may help managers in boosting the quality of their relationships with consumers by enhancing their commitment, trust, and satisfaction. As a result, they are able to retain existing consumers, acquire new ones, and foster long-term relationships with consumers in the digital world.

Finally, relationship quality, such as consumer trust, satisfaction, and commitment, assists managers in increasing consumers’ online repurchase intentions. For example, managers may assist consumers in securing their privacy and offering a trustable online platform for financial transactions, which would directly impact the quality of their relationships with consumers in terms of increasing their trust, commitment, satisfaction, thereby positively influencing their online repurchase intentions. Thus, these strategies may win consumer loyalty in the e-commerce environment. In sum, by employing diverse SMMAs, such as interactivity, informativeness, WOM, personalization, and trendiness, managers can strengthen the quality of their relationships with consumers by increasing their trust, satisfaction, and commitment. Consequently, these factors can increase consumers’ online repurchase intentions in the e-commerce business.

## Conclusion, limitations, and future research scope

This study concluded that SMMAs (interactivity, informativeness, WOM, personalization, and trendiness) significantly influence relationship quality factors, such as commitment, trust, and satisfaction, which in turn positively increase consumers’ online repurchase intentions in China’s e-commerce environment. Further, the mediating role of relationship quality (commitment, trust, and satisfaction) was very important because SMMAs did not directly influence consumers’ online repurchase intentions. Thus, by using SMMAs and relationship quality factors, firms can increase consumers’ online repurchase intentions in China’s e-commerce industry. This study would help the firms in improving consumers’ loyalty and relationships as well as the positive impact on increasing their business volumes in the Chinese e-commerce industry.

This study revealed some limitations that cannot be overlooked. First, this study used SMMAs as a higher-order construct. Future research may examine the influence of each component of SMMAs, such as interactivity, informativeness, WOM, personalization, and trendiness; these factors may shed new insights into the theory and managerial practices. Second, this research used the mediation effects of relationship quality (trust, satisfaction, and commitment) in the e-commerce industry. Future studies may consider the brand’s authenticity in an online context, as brand authenticity is a rapidly growing concept in the current age (Safeer et al., 2021a,c). Third, this study did not examine any moderation effect on the proposed model. Future research may examine by using moderators, such as consumer personality types; such research may help managers to explore various niche market segments depending on consumer personality type. Fourth, this research exclusively focused on Chinese consumers. Although China is an emerging market. However, it would be more insightful if future research examines emerging vs. developed markets to analyze the influence of SMMAs on the e-commerce industry. Finally, this research considered the general (all types) products in order to generalize the findings. Future research may consider specific products/brands with different buying behavior, such as consumer impulse buying behavior (Akram et al., 2017, 2018a,b) in the current topic context.

## Data availability statement

The raw data supporting the conclusions of this article will be made available by the authors, without undue reservation.

## Author contributions

MS identified the research gap, introduction of the topic, and developed the theoretical model and associated hypotheses. AAS and AM worked on methodology, results, discussions, and contributions. All authors read and approved the final manuscript.

## Footnotes

1
https://www.wjx.cn
